# Spheroids as a Type of Three-Dimensional Cell Cultures—Examples of Methods of Preparation and the Most Important Application

**DOI:** 10.3390/ijms21176225

**Published:** 2020-08-28

**Authors:** Kamila Białkowska, Piotr Komorowski, Maria Bryszewska, Katarzyna Miłowska

**Affiliations:** 1 Department of General Biophysics, Faculty of Biology and Environmental Protection, University of Lodz, 141/143 Pomorska St. Building D, 90-236 Lodz, Poland; maria.bryszewska@biol.uni.lodz.pl (M.B.); katarzyna.milowska@biol.uni.lodz.pl (K.M.); 2 Molecular and Nanostructural Biophysics Laboratory, “Bionanopark” Ldt., 114/116 Dubois St., 93-465 Lodz, Poland; p.komorowski@bionanopark.pl; 3 Department of Biophysics, Institute of Materials Science, Lodz University of Technology, 1/15 Stefanowskiego St., 90-924 Lodz, Poland

**Keywords:** 3D cell culture, spheroids, drug testing

## Abstract

Cell cultures are very important for testing materials and drugs, and in the examination of cell biology and special cell mechanisms. The most popular models of cell culture are two-dimensional (2D) as monolayers, but this does not mimic the natural cell environment. Cells are mostly deprived of cell–cell and cell–extracellular matrix interactions. A much better in vitro model is three-dimensional (3D) culture. Because many cell lines have the ability to self-assemble, one 3D culturing method is to produce spheroids. There are several systems for culturing cells in spheroids, e.g., hanging drop, scaffolds and hydrogels, and these cultures have their applications in drug and nanoparticles testing, and disease modeling. In this paper we would like to present methods of preparation of spheroids in general and emphasize the most important applications.

## 1. Introduction

Cells have been cultured since the 1940s [1], and are generally in use to examine cell biology and molecular mechanisms [2]. Cells are taken directly from a tissue and, after suitable preparation, transferred into an artificial environment or they are obtained from a cell line already adopted by others. Cells grow in a medium containing the required nutrients, growth factors, and hormones, in an incubator. Cultures are kept in special dishes placed in strictly controlled temperature conditions, normally a 37 °C [3]. Cells are attached to a flat surface as a substrate, glass or plastic, mainly in two dimensions, as monolayers. This method of cell culturing is most popular because it is simple and convenient; it has been an invaluable method providing important knowledge as models of variety diseases [4,5]. However, forcing cells to grow on flat surfaces can change their metabolism and functioning [4]. In 2D cell cultures, the cell–cell and cell–extracellular matrix interactions are reduced, and the level of cellular responsiveness is limited [2,6]. Moreover, cell culture environment can have an effect on the phenotype of cells and hence affect the cellular response to added substances, e.g., drugs [1]. All cells in the body live in 3D environment, which is crucial for their metabolism and growth. The phenotype and functions of each cell are highly dependent on elaborated interactions with neighboring cells, the extracellular matrix (ECM) and proteins [6]. Those cell–cell and cell–ECM interactions differ from 2D to 3D cultures and also between cell layers in spheroids structures, and this can affect cytotoxicity results [7]. For these reasons, testing the toxicity of materials and substances on 2D cell cultures is not exactly predictive of that which might be expected in the body [6,8]. 3D cell cultures more precisely mimic the natural cell microenvironment. The morphology and physiology of cells in 3D cultures are different from cells in 2D cultures, showing responses that correspond in some ways more like in vivo behavior [8]. In 2D models, molecules can be secreted into the culture medium, and, therefore, changing the medium will remove these substances and might disturb some analysis. For example, in 2D models of Alzheimer disease, removing the medium will mean that secreted amyloid beta (Aß) is discarded and, therefore, change the analysis of Aß aggregation. 3D cell cultures can limit the diffusion of Aß into the culture medium [5].

Three-dimensional cell cultures are widely used in investigations of cancer cells, intracellular interactions and cell differentiation, evaluation of substance toxicity and efficacy of potential drugs [9], and therefore show promise in filling the gap between 2D culturing and experiments with animals [10]. It has been shown that 3D cell cultures exhibit increased levels of tissue-specific markers, regain tissue-specific functions and have various profiles of gene expression compared to 2D cultured cells [11]. The authors compared 3D and 2D MCF-7 human breast cancer cells, and showed that cells cultured in 3D systems had a higher mRNA expression of the luminal epithelial markers keratin 8 and keratin 19, and a lower expression of basal marker keratin 14 and the mesenchymal marker vimentin [11]. The 3D spheroids, as in solid tumors, have permeability barriers through which some substances or agents under test have to penetrate [12]. Table 1 shows the most important differences between 2D and 3D cell cultures.

There are a number of formats and materials available that help the culturing of cells in 3D. For example, there are some hydrogel substrates, e.g., beads, injectable gels, moldable gels, and macroporous structures. Other techniques are also available that help prepare 3D cultures, e.g., hanging drop, low-binding plastic, pyramid plates. There are also macroporous scaffolds, e.g., meshes, foams or fibrous patches that allow spatial organization of cells and seeding cells throughout the thickness of the matrix, but they have cell-matrix interactions closer to 2D cell cultures (and are therefore called semi-3D or 2.5D cultures). This is characteristic of polystyrene-based 3D cell culture materials [4].

Polymer hydrogels seem to be suitable for 3D cell cultures because of their similarity to physiological extracellular matrix. Synthetic materials that could be applied as hydrogels are polyethylene glycol (PEG), polyvinyl alcohol (PVA), poly(hydroxyethyl methacrylate) (polyHEMA), and polycaprolactone (PCL). Furthermore, natural polymers and proteins can form hydrogels, e.g., alginate, collagen, chitosan, hyaluronan, dextran, and fibrin. Alginate hyaluronan (a product of bacterial fermentation) and dextran are non-animal derived materials [4].

In this paper we would like to outline the methods of spheroid preparation and focus on the most important applications.

## 2. Spheroids as a Type of 3D Cell Cultures

### 2.1. Spheroids

**Figure 1:**
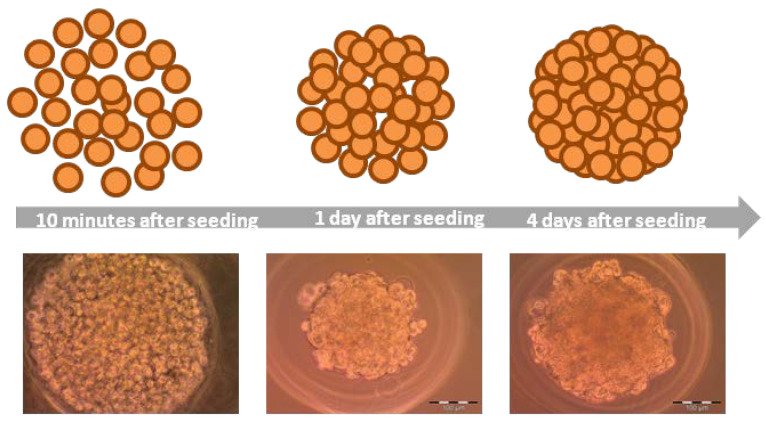
Formation of spheroids on MCF-7 cell line.

Spheroids (Figure 1) are cell aggregates, self-assembling in an environment that prevents attachment to a flat surface [9,12]. Spheroid formulation is possible because of membrane proteins (integrins) and extracellular matrix proteins [9]. During spheroids formation three steps could be defined: (i) dispersed cells aggregate due to long-chain ECM fibers consisting RGD motifs that allow to bind cell-surface integrin and this leads to upregulated cadherin expression, (ii) cadherin accumulates on the surface of cell membrane, (iii) the hemophilic cadherin–cadherin binding between neighboring cells allows to tighten connections between cells and spheroids are formed [14,21]. Moreover, integrins are involved in activation of focal adhesion kinase (FAK), which is a cytoplasmic tyrosine kinase. Invasive phenotype of tumor, increased tumor growth, and poor patient prognosis are associated with overexpression of FAK. Knockout of FAK in mouse tumor models leads to prevention of some aspects of initiation and progression of breast carcinoma tumor [22]. FAK is involved in cell adhesion, migration, and also growth. FAK influences the rearrangement of the cytoskeleton (actin filaments) and microtubules, and this affects cell adhesion and migration. Moreover, FAK transmits extracellular signals associated with integrins [23]. Cytoskeleton proteins are responsible for the mechanical integrity [9]. Actin cytoskeleton is crucial in adhesion, mediation of cell shape, migration, and spreading. Furthermore, actin skeleton plays an important role in spheroids formation. Blocking polymerization of actin filaments reduces aggregation of T47D, HC11, and 4T1 cells strongly. Microtubules also take part in cell aggregation and the growth of spheroids. Interference with the polymerization of microtubules slows down the aggregation of cells or results in the decrease of compaction of spheroids in HC11 cells [23]. Oxygen supply to 3D cell culture is a very important factor limiting cell viability during culturing cells. Anada et al. (2012) showed that after 10 days in culture, spheroids stopped growing on non-oxygen-permeable chips and the diameter remained constant at approximately 360 μm. They tested 3D culture chips made of gas-permeable polydimethylsiloxane (PDMS) and noticed, that after 14 days in culture, spheroids on those PDMS chips continued growing until approximately 600 µm [24]. During culturing, cells may be of different sizes; cells within spheroid are smaller than cells on the outside [9]. Cells remaining on the periphery of the spheroid proliferate more actively [25]. Several techniques are used to form spheroids.

#### 2.1.1. Hanging Drop

**Figure 2:**
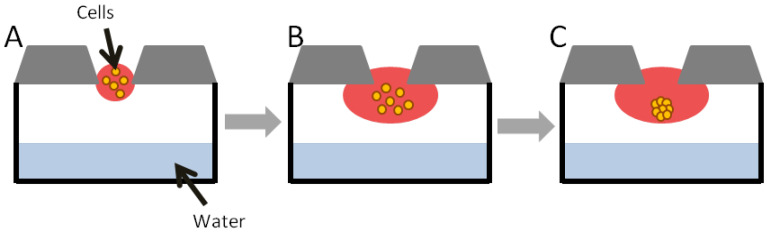
Spheroid formation in hanging drop method: (**A**)—cell suspension dispensed; (**B**)—cells in hanging drop; (**C**)—cells aggregate to form spheroid [29].

Hanging drop (Figure 2) is one method of obtaining scaffold-free cell cultures [11]. This technique has some limitations, including low throughput, spherical geometry, and a high shear force environment [26]. Some manipulations, e.g., changing the medium and adding of compounds can be complicated and time-consuming [11]. Some cell lines do not form compact spheroids using this method [25]. This method, however, does not require specialized equipment [27], but involves small volumes of cell suspension (usually 20 µL). Cell density depends on the required size of the spheroid. The cell suspension can be placed into the well of a special plate, which is turned upside down so that the cell suspension becomes a hanging drop held by surface tension [28]. Cells remain in direct contact with each other and with the ECM [27].

The simplest way to obtain cell culture in hanging drop is to put a drop with a cell suspension onto the inside of a lid of a culture plate. After reversing, microgravity concentrates cells at the bottom of the drop [9]. The hanging drop method could be used also to co-culture several cell lines [27].

Tung et al. (2010) described a hanging drop culture plate in 384-well format that can be adapted to high-throughput screening (HTS) instruments available to 2D cultures, e.g., liquid handling robots. The plate is made of polystyrene and contains 16 rows and 24 columns. On the edges of the plate a water reservoir is located. When it is filled with water it avoids an evaporation. A small volume of culture media causes its rapid evaporation and changes in its osmolality, which should be stable during long period of cell culturing. For this reason, the 384-hanging drop array plate is surrounded by a plate lid from the top and by 96-well plate filled with water from the bottom. On the top of every well of the 384-hanging drop array plate there is an access hole through which cells are seeded and medium is exchanged. During culturing the whole system is wrapped with Parafilm. To obtain a spheroid, 15 µL of cell suspension was added to access hole to create a hanging drop. To change a medium 5 µL of liquid was taken and then 7 µL of a fresh growth medium was added [30].

Osmolality of culture media was investigated, and the results showed that it was in the optimal range of 300 to 360 mmol/kg. The authors used cell lines: African green monkey kidney fibroblasts (COS7), murine embryonic stem cells (ES-D3), and human epithelial carcinoma cells (A431.H9) that stably express mesothelin [30].

A431.H9 cells in hanging drop cell culture were treated with 2 types of anticancer drugs and the viability was measured. After incubation with tested drugs alamarBlue was added and fluorescence was measured using plate reader. Due to the possibility of using liquid handling robots and popular plate readers, the 384-hanging drop array plate enables to perform HTS [30].

#### 2.1.2. Hydrogels

Among the systems for spheroid culturing there are non-adhesive agarose hydrogels that do not have the influence of an ECM. This type of cell culturing has some advantages, ease of maintenance, the possibility of controlling the microtissue size, and a large amount of microtissues per plate [11]. In this technique, cells are seeded on hydrogel with recesses where cells sink and can self-assemble into 3D spheroid microtissues. Without any influence from the ECM, cells in homogenous suspension can self-assemble spheroids, and cells in heterogeneous suspension self-segregate and form multilayered structures [26]. Napolitano et al. (2007) cultured different types of cell lines to show the versatility of the technique using MCF-7 human breast cancer cells, human umbilical vein endothelial cells (HUVEC), normal human fibroblasts (NHF), rat hepatoma cells (H35) and rat glioblastoma cells (RG2). With the aid of computer-assisted design, they created special molds including a cell-seeding chamber, recesses for cell aggregation, and ports for exchanging medium. Those micromolds were filled with sterilized agarose to form the right substrate for cell culturing [26]. Because the substrate was non-adhesive, cell-to-cell binding was favored, and cells self-assembled in spheroids. These cell-to-cell interactions were maximized because of the shape of the bottom hydrogels. They showed that use of hydrogel gives versatility for controlled microtissue production [26]. Some cell lines require ECM proteins in culture medium to create shapely spheroids [9].

Some micromolds for preparation of hydrogels are commercially available. For example, Vantangoli et al. (2015) used them to prepare agarose hydrogel, for MCF-7 cell testing [11].

#### 2.1.3. Rotary Cell Cultures

**Figure 3:**
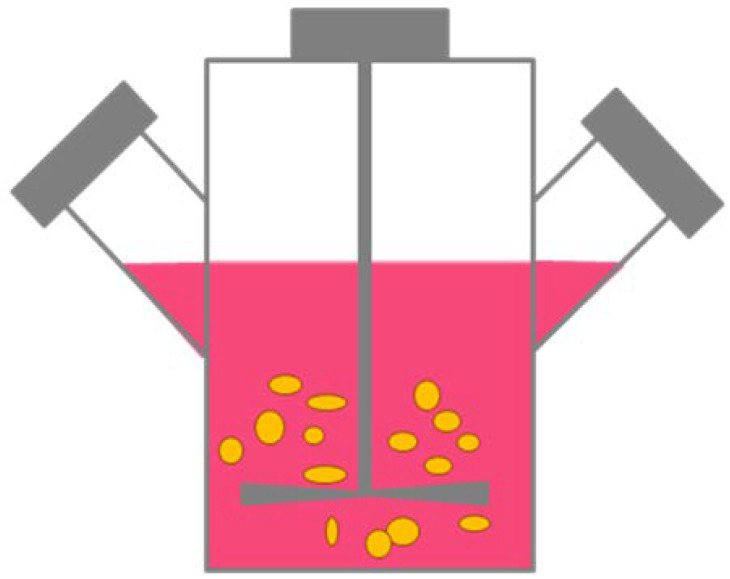
Cell culture in bottle with agitator. Cells start aggregating and self-assembling [9].

One of the methods of obtaining spheroids is cell culture in a bottle with an agitator (Figure 3). In these conditions, cells cannot attach to the substrate, and start aggregating and self-assembling. It is one of the simplest methods to produce spheroids on a large scale. This method has certain disadvantages e.g., longevity of cultures, variation in spheroid size, and mechanical damages of cells. One variation of this method is a system with a flask rotating around a horizontal axis. Simulation of microgravity with minimal hydrodynamic forces does not destroy cells, such that this method allows the formation of bigger spheroids than in a bottle with an agitator. Morphological differences between spheroids are also smaller than in the first method [9].

#### 2.1.4. Cell Suspension with the Addition of Nanofibers

This method of producing spheroids is by the addition of polymer nanofibers to a suspension of adherent cells. Shin et al. (2012) added poly(lactic-co-glycolic acid) (PLGA) nanofibers to a suspension of human embryonic kidney 293 cells (HEK) and human dermal fibroblasts (HDFs), although this could be for all types of adherent cells [31].

Nanofibers increase spheroid production and reduce cell death due to cell non-adherence. In a cell suspension lacking nanofibers, neighboring cells interact because of cadherins. When nanofibers are added, spheroid formation is promoted also by the interaction of cells with them [31]. Cell binding to nanofibers may be due to the action of vitronectin and fibronectin from the serum in the medium [32]. Those proteins when added to the culture medium adsorb on the nanofibers and then cells attach to those nanofibers forming spheroids [31].

#### 2.1.5. Magnetic Levitation Method

Magnetic levitation is one of the methods to produce scaffold-free 3D cell cultures. Thanks to magnetic levitation cells associate into 3D cell culture and produce ECM, keeping cellular activity. In this technique the magnetic force overcomes the gravitational force [33]. Cells are treated with paramagnetic iron oxide nanoparticles overnight, which allows for their uptake by cells. Cell culture is washed and then treated with trypsin solution and seeded into low-adhesive plates. Finally, the magnet is placed on a top of the plate lid that leads to pulling labeled cells up under magnetic forces. Spheroids are created within few hours [29].

Türker et al. (2018) investigated the levitation platform using gadolinium(III) chelates (GD(III) chelates), which are paramagnetic agents. They suspended cells in capillary channels and the cell medium was paramagnetized using various Gd(III) chelates. After placement, the capillary channel into the magnetic levitation platform the cells levitated to a levitation height (z) or equilibrium height. Because the environment was paramagnetized, the cells reached the levitation height, migrating from a higher magnetic field region to a lower magnetic field region. The 3D cell culture formation took place at the levitation height, where the cells interacted and assembled [33]. The authors tested viability of the NIH 3T3 mouse fibroblasts after incubation with three types of gadolinium agents (Gadobutrol, Gadoteric acid and Gadodiamide) using MTT and Live/Dead assays. The data showed that cells after treatment with Gadobutrol exhibited higher viability compared to other agents [33].

Souza et al. (2010) proposed a model of 3D cell culture using magnetic levitation combined with hydrogels with gold and magnetic iron oxide (MIO) nanoparticles and filamentous bacteriophage. Hydrogels were obtained via mixing the solution of gold nanoparticles with MIO nanopowder (magnetite [Fe_3_O_4_]). Then, the solution was mixed with phage solution of equal volume and, finally, put at 4 °C overnight, to allow to form hydrogel. Preparing levitated cell culture consisted of treatment of the cells with hydrogel (1 μL/1 cm^2^ of surface area) and incubation overnight. Then cells were detached using trypsin-EDTA solution in PBS and seeded into a culture Petri dish. The cell line used in this study was human glioblastoma cells, genetically modified. Cells were grown for 8 days, and during this period the fluorescence of mCherry protein was observed confirming the viability of the cells in the 3D structures. Within 30 min. after seeding, the cells were collected together. Spheroids were obtained after 3–8 days [34].

Souza et. al. (2010) investigated molecular similarity of 3D cell cultures obtained using magnetic levitation to orthotopic human tumor xenografts from immunodeficient mice. They measured expression of transmembrane protein, N-cadherin. The expression of this protein in the 3D culture showed that magnetic levitation exhibits some features similar to the in vivo model. This suggests that 3D cell cultures based on magnetic levitation method could be a cheaper substitute for expensive and labor-intensive method based on human tumor xenografts from immunodeficient mice [34].

#### 2.1.6. Microfluidic Systems

The described methods are non-microfluidic methods. They play an important role in the formation and investigation of spheroids, but they have also some limitations. Some disadvantages of these methods (e.g., hanging drop method) are differences in spheroids’ diameters, low-throughput, or labor intensity. The non-microfluidic environment causes reduction of oxygen and nutrients and the increase of osmolality and level of metabolites. To overcome those limitations the microfluidics systems are created. The device with microfluidic flow is made of microwells which are connected by microfluidic channels, from simple to more sophisticated arrays of microchannels. Microchannels are prepared through etching or forming on the surface of using neutral materials, e.g., silicon, glass or polydimethylsiloxane (PDMS). The cells are cultured above layers made of matrix coated porous membrane and with direct contact with endothelial cells. Additionally, immune cells and tumor cells flow through the microchannels [29].

Very important advantages of microfluidic systems are controlled mixing, chemical concentration gradients, lower consumption of reagents, control of shear stress and pressure on cells, and also constant perfusion. Microfluidic chips provide dynamic environment for better reflection of tissue environment [7]. The sizes of spheroids are homogenous [35]. Viability of hepatocytes cultured in spheroids in flow conditions is higher than in static model [7,36]. Cancer spheroids cultured in a microwell plate in dynamic conditions exhibit higher resistance for drugs than in no-flow conditions [7,35].

#### 2.1.7. Spheroids Based on Co-Cultures

The tumor microenvironment is heterogenous; therefore, the biology of tumor is the result of mutual influence between cancer cells and their environment. For example, fibronectin, one of the ECM protein, takes part in the regulation of tumor stiffness, promotes the growth of the tumor and resistance for drugs. Interactions between cancer cells and surrounding fibroblasts and also immune and endothelial cells are connected to regulation of tumor progression. Fibroblasts are involved in metastasis of the tumor and also in tumor development. During tumor vascularization the migration and the proliferation of endothelial cells takes place and they depend on interactions between ECM proteins, fibroblasts, and cancer cells. Such complex systems need reflection in an appropriate in vitro model, to better mimic tumor environment [37]. For this reason, Lazzari et al. (2018), proposed a spheroid model of pancreatic tumor, based on a triple co-culture of pancreatic cancer cells (PANC-1) together with fibroblasts (MRC-5) and endothelial cells (HUVEC). The authors proved that in complex environment cancer cells are less sensitive to chemotherapy [37].

Xin et al. (2019) distinguished two platforms for 3D cell co-cultures: co-cultures with the cell-cell contact and co-cultures without the cell-cell contact. Co-cultures with the cell-cell contact allow to evaluate interactions between cancer cells and stromal cells, mediated by adhesion. Among those methods there is a co-culture with direct contact, where cancer and stromal cells are mixed to form heterogonous spheroids, and co-culture with the semi-contact, where homogeneous cancer cells spheroids are seeded into 3D scaffolds combined with stromal cells. Cells in non-contact co-cultures are not allowed to contact and adhere together, because they are kept in distinct layers or chambers. In those methods interactions between cancer and stromal cells could be assigned to chemical mediators [38].

#### 2.1.8. Bioprinting

Simple structure and low vascularization potential are the main limits in using current 3D cultures models. The lack of vascularization limits the spheroids size and probably does not mimic later stages of tumors very well. In most of the models of 3D cultures the spatial distribution of cancer cells and ECM composition is not well arranged [39]. The solution to lack of vascularization and designing scaffolds for better reflection of tumor microenvironment and heterogeneity could be bioprinting technology [40]. Bioprinting includes variety of approaches consisting of distributing of biological materials and cells in a spatially defined way [39]. In other words, the bioprinting technique could be defined as a technology in which cell layers and supporting biological materials are positioned precisely to mimic functions of the tissue or organ [41]. There are few strategies of bioprinting: inkjet printing, extrusion-based printing, laser-assisted printing and stereolithography [39]. In the inkjet bioprinting the droplets made of bioink (cell-laden) filling the cartridge are generated and deposited on a scaffold precisely. A computer program controls the deposition of the droplets and it leads to the creation of 3D structures. This method is relatively fast and not expensive and provides high viability of cells [39,40]. In the extrusion-based bioprinting the bioink is moved through the nozzle with pneumatic or mechanical pressure. This method is also quite cheap and fast. In the laser-assisted bioprinting laser stimulation leads to a response of the donor layer which absorbs energy and generates a high-pressure bubble that leads to pushing a droplet of the bioink onto the substrate. Some disadvantages of this method are expensive equipment and poor choice of bioinks. In the stereolithography an array of programmed mirrors generates a digital mask, which is projected to reservoir with bioink to photo-crosslink patterns layer by layer. This method is characterized by high resolution and fast speed [39].

## 3. Applications of 3D Cell Cultures

### 3.1. Drug Testing and Nanoparticles Examination

#### 3.1.1. Drug Testing

A generation of new cancer drugs is based on three approaches, referred to as: (i) high throughput drug screening (HTS), (ii) expansion of analogs of existing drugs, and (iii) rational drug design. These involve assays based on measuring cell viability, proliferation, and clonogenicity in an in vitro environment [42]. Cell cultures help assess drug safety and indicate their possible mechanism of action. Test substances are added to the culture medium and their activity investigated [43]. Presently, 2D cell cultures remain very useful in drug investigation. However, as already mentioned, 2D models do not mimic well the physiological environment of living cells [34]. For instance, cells of the colon cancer cell line, HCT-116 wt, cultured as spheroids were more resistant to some of the tested drugs compared with them cultured as a monolayer [42,44].

Cells grown in 3D cultures can be maintained longer than as 2D monolayers. The 3D aggregates can be kept for 4 weeks, whereas cells in 2D cultures last approximately 1 week before reaching confluence. For this reason, 3D cell cultures make a better model for studying long-term effects of drugs. Tumor cells in a monolayer proliferate faster than in 3D aggregates and are more sensitive to agents used during chemotherapy or radiation therapy [3].

Karlson et al. (2012) used 96-well NanoCulture^®^ plate to form spheroids from the human colon cancer cell lines HCT-116 wt, HCT-116 wt/GFP and HCT-116 HRP EGFP (hypoxia-responsive promoter enhanced green fluorescent protein) cell lines. 3- and 6-day spheroids were tested, using standard drugs in the treatment of colon cancer, including 5-FU, oxaliplatin and irinotecan (Table 2). They also used melphalan (Table 2), used clinically in treating some cancers. They also used topoisomerase inhibitors, acriflavine, and VLX50, which is now in an early phase of evolution. Cells were incubated for 72 h with each drug. After preliminary experiments on monolayers, these authors selected three suitable concentrations of each drug. HCT116 wt and HCT116 wt/GFP cell lines cultured as monolayers were equally highly sensitive to 5-FU, oxaliplatin, irinotecan, and melphalan, which indicated that GFP-labeling does not influence the phenotype. For this reason, GFP-labeled cells were used to following experiments on drug cytotoxicity. The results showed that 3-day old spheroids were more resistant to four standard drugs and 6-day old spheroids were almost totally resistant to these drugs. The fact that cells cultured in 3D systems were more resistant to these drugs is closely related to geno- and pheno-typical changes caused by spheroid formation [42].

Koban et al. (2018) tested gefitinib (Table 2), a specific inhibitor of the epidermal growth factor receptor (EGFR), and used as a treatment for non-small-cell lung cancer. They used it as antiviral compound for treating primary human keratinocytes (NHEK) kept in a 3D ECM-based cell culture. The results were then compared to the effects on analogous 2D cell culture [19]. The morphology of NHEK cells grown in 3D systems more closely mimic in vivo physiology than traditional monolayers. In 2D cell cultures, gefitinib showed antiviral activity at concentrations too high for in vivo application [14,45]. Significant reduction of virus replication occurred at 25 µM gefitinib, which was cytotoxic. In 3D cell culture, 0.5 µM gefitinib was sufficient to induce a clear reduction of the replication and spread of the virus. Near total inhibition of viral replication and EGFR phosphorylation were reached at 5 µM, without any obvious cytotoxic effects [19].

Orthopoxviruses (OPV) are double-stranded DNA viruses which replicate in host-cell cytoplasm. Virus spread and replication are supported by some proteins encoded by OPV, e.g., growth factor proteins. One of these proteins, epidermal growth factor (EGF)-like protein, activates EGFR, which enhances host cell proliferation and inhibits apoptosis [19].

The reason that viral replication and also cell proliferation at low concentrations of gefitinib were lower in 3D cell than in 2D systems might be that proliferation in the former is generally much less, even in non-infectious conditions. Viral infection may be an impulse for cell proliferation due to expression of the EGF homologue cowpox growth factor (CGF). Expression of this factor is significantly higher in infected cells in 3D models than in 2D models 48 h post-infection (p.i.). Moreover, expression of EGFR on the surface of cells in 3D cultures is clearly lower than on their counterparts in 2D culture [19,46]. As a result, in 3D cultures inhibition of only a small part of EGFR on the cell surface could be stronger and more crucial in reducing cell proliferation than in 2D cultures [19].

Perut et al. (2018) compared the effects of anticancer drugs on human chondrosarcoma (SW1353) and osteosarcoma (MG-63DXR30 cell line obtained from parental MG-63) cell lines [20].

Chondrosarcoma is a bone (cartilage) sarcoma of adults. It is resistant to chemotherapy and radiotherapy, and is, therefore, treated only by surgery [20,47]. Resistance is probably caused by the poor proliferation potential [47]. New drugs are now being tested that target this tumor. Several possible targets have been discovered, but this has failed to develop effective therapies for patients [48]. Failure in the search for rewarding therapy probably is a result of weakness of the monolayer tumor model—2D cell cultures do not mimic tumor structure adequately [20]. Perut et al. (2018) used a spheroid cell culture (hanging drop method) to test several anticancer drugs, anti-mitogenic DXR, cisplatin (CIS) and salinomycin (SAL; Table 2). They have investigated the anti-autophagic chloroquine (CQ) because of reports suggesting a role for autophagy in tumor resistance [20,49]. Autophagy is a degradation process of proteins and organelles, which can be reused to by cell [50].

SW1353 cells grown in 2D and 3D cell cultures were treated with DXR and CIS. After 72 h, cell viability in spheroid cultures was higher than in 2D cultures, with similar effects being seen with CQ. The only drug which gave similar results for monolayers and 3D cell cultures was SAL [20].

Resistance of 3D chondrosarcoma cultures to anticancer drugs could also be explained by a characteristic structure of 3D culture. In the 3D spheroid, an important role is played not only by cell-cell interactions, but also by internal or external biochemical signals that are part of the tumor microenvironment, e.g., hypoxia, limited access to nutrients, or acidosis [20]. As chondrosarcoma is a tumor with rather low vascularity and is highly hypoxic, it is important to establish low pH and low oxygen tension conditions [20,51]. Perut et al. (2018) measured the expression of a marker of hypoxia, CA IX, and found that the level was remarkably higher in spheroids than in monolayers [20].

#### 3.1.2. Nanoparticle Examination

Testing of nanomaterials and their effects on cells is important because of possible therapeutic application. Nanomaterials are considered as safe gene carriers in gene therapy. Gene therapy is a potential method for fighting diseases, such as cancer, when traditional treatment is poor [52].

**Figure 4:**
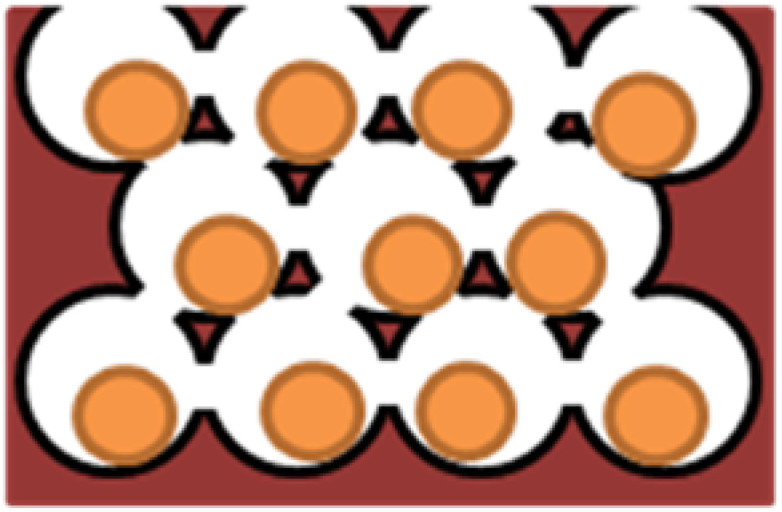
Model of hydrogel inverted colloidal crystal (ICC) scaffold [6].

Because of the lack of the nanoparticle (NP) transport through cell layers in 2D cell cultures, 3D cell culture offers a better model. Techniques used for testing nanoparticle toxicity are the same as the methods for drug examination, although the toxic mechanism can be different [6]. Lee et al. (2009) have introduced 3D spheroid-culture-based NP toxicity testing system (Figure 4) using human hepatocarcinoma (HepG2) cells, because the liver is the main organ for NP accumulation. As a substrate, they used transparent and nonadhesive polyacrylamide hydrogel to measure the toxic effects of cadmium telluride (CdTe) and gold (Au) nanoparticles. Morphology, metabolic activity, membrane activity, and mechanism of cell death were explored, comparing the results from 3D cultures with those from 2D cultures. Cell number and spheroid diameter were crucial parameters to get repeatable results. They also showed that the activity of a spheroid depends on its size. They found significant differences between the morphology of cells in 2D and 3D cell cultures after treatment with CdTeNPs, with more death in 2D than in 3D cultures. Cell toxicity assays also confirmed that the toxic effects of NPs were reduced in 3D compared to 2D cultures [6].

Zeng et al. (2016) examined the effects of polyamidoamine (PAMAM) dendrimers on human neural progenitor cells in 3D neurosphere systems imitating the nervous system. Cells were treated with dendrimers from G4 group at 0.3, 1, 3, and 10 lg/mL. Fluorescent-labeled dendrimers aided measurement of their biodistribution, and microarray analysis were used to investigate gene expression [53].

Dendrimers are highly branched, synthetic molecules of spherical shape [54,55]. PAMAM dendrimers have a characteristic structure with a 2-carbon ethylenediamine core surrounded by functional groups [53]. These authors demonstrated that PAMAM dendrimers could get through external cells of neurospheres and penetrate them. Some groups of dendrimers inhibited cell proliferation and neuronal migration. They found 32 genes related to toxicity caused by dendrimers [53].

Goodman et al. (2007), evaluated the impact of nanoparticle size and collagenase treatment on the diffusion of carboxylated polystyrene nanoparticles into spheroid cell culture [56]. ECM is considered a factor involved in the resistance to therapeutic agents because it prevents penetration into the tumor [57]. Diffusion of molecules depends on tumor type and its localization [58]. Penetration of particles (such as viruses) increases after injections of protease enzymes into tumors. Immobilization of collagenase on the surface of nanoparticles leads to digestion of ECM proteins and also increases delivery of nanoparticles into a spheroid [56].

According to Goodman et al. (2007) particles <100 nm penetrate poorly into a spheroid core. Treatment of spheroids with nanoparticles coated with collagenase influences penetration of smaller nanoparticles (up to 100 nm) more than particles >100 nm. This means that coating nanoparticles with enzymes degrading proteins of the ECM could improve delivering them into solid tumors [56].

### 3.2. Models for Neurodegenerative Diseases

The 3D cell cultures are widely used in medical studies [3], e.g., research on neurodegenerative diseases [59].

Neurodegenerative diseases are a group of congenital or acquired disorders of the nervous system, characterized by progressive degeneration of neural cells, leading to their death. Neurons show pathological changes resulting in the formation of aggregates of modified proteins that are neurotoxic and resistant to proteolytic enzymes. Among these abnormal proteins are ß-amyloid (Aß) in Alzheimer’s disease (AD), α-synuclein in Parkinson’s disease (PD), and the huntingtin protein in Huntington’s disease (HD). Those proteins disturb the functions of neurons and eventually lead to necrosis or apoptosis [59,60,61].

#### 3.2.1. Alzheimer Disease

The most widespread neurodegenerative disease in the world is Alzheimer Disease (AD), for which there is no effective therapy, only some symptomatic treatment [62,63]. It is characterized by a progressive cognitive decline and involves memory deterioration. Orientation, judgments, and reasoning are also disturbed [64]. There are two characteristic features of AD, namely plaques of ß-amyloid and neurofibrillary tangles of tau protein [59,63,65]. Aß is generated from amyloid precursor protein (APP) during the process caused by two enzymes, ß-secretase and γ-secretase [62]. The hypothesis that Aß accumulation is the initial event in AD, leading to the next pathological events is called the “amyloid cascade hypothesis” [66].

Transgenic mice have now been used as models for studies on AD, but unfortunately, they do not exhibit important features occurring in humans [63]. Additional phenotypes of mice can also occur, which are not related to AD [67]. For these reasons, therapies for AD that are effective in mouse models probably do not work on humans [63,68]. In transgenic mice, there is also no amyloid cascade [66,69]. According to Choi et al. (2016) the Matrigel-based 3D cell culture system is a more appropriate model for AD testing as Aß plaques are present, and these are not present in the mouse model [63].

SH-SY5Y is a neuronal-like cell line that is artificially differentiated to neural cells. This cell line came from the bone marrow of a patient with neuroblastoma [59]. Characteristic features of the cells are activities of dopamine-ß-hydroxylase and tyrosine hydroxylase, some level of noradrenaline (NA) release, and the presence of choline acetyltransferase, acetylcholinesterase, and butyrylcholinesterase [70]. Seidel et al. (2012) used spheroids of human neuroblastoma cell line (SH-SY5Y) which overexpress EGFP-fused tau as a model to study the pathologies of tau protein in AD. They obtained 3 variants of SH-SY5Y over expressing tau (0N4R), namely wild type (WT), a variant with single point mutation P301L (which is used in common) and K280q (which is 4-fold gene mutation in the tau protein gene DK280, P301L, V337M, R406W), which was used to enhance tauopathy. Generally, differentiation of SHSY5Y cells took place by using several agents, e.g., phorbol esters and retinoic acid, growth factors (like brain derived neurotrophic factor, BDNF), nerve growth factor (NGF) or cholesterol [71]. However, differentiation agents influence cell metabolism and could probably affect the induction of tauopathy. The 3D cell cultures might help in eliminating the problem with differentiation agents [72].

#### 3.2.2. Parkinson Disease

Parkinson disease (PD) is a neurodegenerative illness characterized by a loss of cells in the substantia nigra in the midbrain. The loss of these dopaminergic neurons is related to motor dysfunction [73,74], resting tremors, bradykinesia, postural instability and rigidity [75,76]. There is no representative in vitro model to study this neurodegenerative dysfunction. Animal models are not sufficient to predict responses occurring in humans [73]. Since there is the possibility of obtaining most major cell types from the human brain during differentiating induced-pluripotent stem cells—iPSCs [77], this research model seems to show promise as an accurate human model for PD [73].

**Figure 5:**
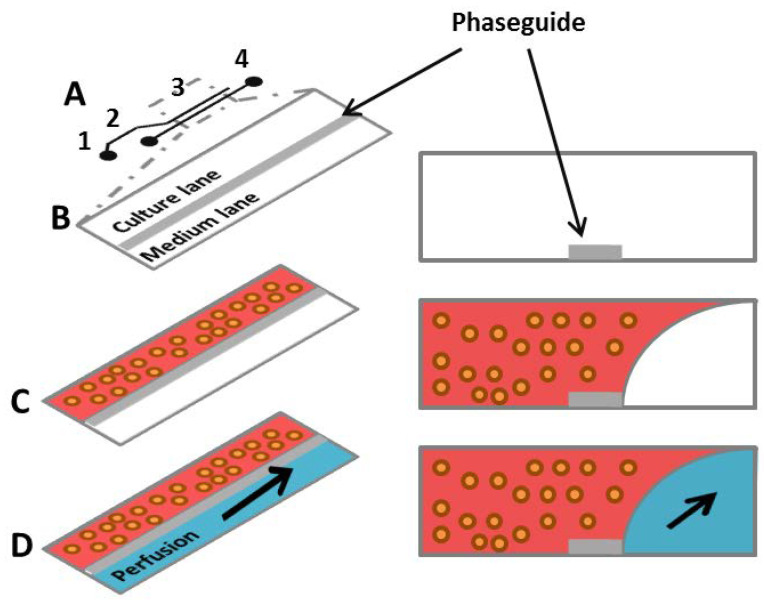
(**A**) A single 2-lane bioreactor scheme composed of: 1—a gel inlet; 2—a perfusion inlet; 3—an optical readout window; 4—a perfusion outlet; (**B**) the readout window and its cross section (horizintal view); a phaseguide separates a 2-lane chamber and allows to selective gel patterning; (**C**) melted gel with cells is loaded and selectively patterned by the phaseguide; (**D**) after gelation the medium is provided in the perfusion lane and gravitational leveling leads to perfusion between the perfusion inlet and the perfusion outlet wells [78].

Moreno et al. (2015) obtained human neuroepithelial cells from iPSCs and finally differentiated them as receiving dopaminergic neurons, cultured within 3D microfluidic cell culture bioreactors. After 30 days, those neurons had characteristic features of dopaminergic neurons and were active [78]. The 3D culture bioreactors were described by Trietsch et al. (2013), who proposed a platform that mimics tissue and perfusion excluding spatial separation [79]. Neighboring lanes of gels and liquids reproduced tissue heterogeneity. A single bioreactor is made from a row of cells settled in hydrogel, and one or more neighboring lanes of liquid flowing laminarly (Figure 5). To shape liquids flowing into the bioreactor, each pair of lanes is separated using a phaseguide. Phaseguide technology makes it possible to control the filling and emptying of a range of types of microfluidic constructions [80,81]. Cells are mixed with replacement ECM, which is subsequently distributed into a well plugged to the phaseguide delimited lane. Finally, the fresh portion of medium is added to the well, which is combined with the medium lane neighboring with cells in hydrogel [78]. Moreno et al. (2015) confirmed that using this technique allows them to obtain dopaminergic neurons and proved its usefulness in calcium imaging and immunofluorescence. Moreover, analysis of 3D images showed neurons with long neurites [78].

### 3.3. Hepatocyte Spheroids as A Model for Studying Liver Functions and Diseases

Primary human hepatocyte (PHH) spheroid system is a promising tool to investigate liver diseases, functions, long-term drug-induced liver injury, and drug testing, since monolayers seem to be useless due to their rapid de-differentiation. Culturing PHH spheroids in serum-free and chemically specific conditions makes them similar to liver in vivo. Furthermore, some inter-individual variability could be observed. Moreover, morphology, viability, and some functions specific for hepatocytes could be noticed after a minimum 5 weeks of culturing. Spheroids remain phenotypically stable. PHH cells could be co-cultured with non-parenchymal cells e.g., Kupffer cells and biliary or stellate cells and this supports their long-term viability [82].

Bell et al. (2016) performed proteome analysis of PHH cells cultured in spheroids (7 days spheroids) as described above and cells from the same donor cultured as monolayers (after 24 h and 7 days) with livers from which they came from. The rapid changes were observed in 2D monolayers cultures. Measurements after 24 h showed that expression of 457 proteins was changed. After 7 days the differences in expression were seen for 358 proteins and expression of 282 of them were changed also after 24 h. After spheroids measurements it was observed that fewer proteins showed altered expression. Moreover, in spheroids cells retained inter-individual differences, what was proved when compared to the corresponding liver pieces from which they were obtained [82].

Analysis of albumin secretion exhibited that hepatocyte-specific functions in the PHH spheroid were kept during prolonged culture, and the secretion was stable [82].

PHH spheroids seem to be a good model for testing liver pathologies. Exposing spheroids to chlorpromazine led to notable accumulation of bile acid, which suggested disturbances in bile acid transport characteristic for cholestasis. Moreover, treatment of the PHH spheroids with cyclosporine A caused increase of neutral lipids which is associated with steatosis. This indicates that the PHH spheroid model could recreate steatotic pathologies in vitro. Furthermore, this model is appropriate for studying the underlying mechanisms of this disease and for drug screening [82].

## 4. Conclusions

The 3D cell cultures seem to be a suitable tool to improve on the imitations of the simpler 2D cell cultures, which do not simulate the physiological environment precisely as studies on animals. The 3D culture models have the potential for drug testing and discoveries and the examination of nanoparticles. They could also be used as models for diseases, e.g., neurodegenerative diseases or tumors, since animal models do not have some of the relevant and important features that are found in humans, which limit applicability.

The 3D cell cultures offer more in cell–cell and cell–ECM interactions compared to the more traditional use of monolayers (2D cultures), and can have structures more similar to those found in vivo.

## Figures and Tables

**Table 1:** Comparing of 2D and 3D cell cultures.

2D	3D
• Cell-cell contact is limited [13]; • Cell-flat, plastic surface contact is dominating [9];	• Cell-cell contact is dominating [14,15];
• Contact with ECM only on one surface [9];	• Cells remain in contact with ECM [14,15];
• No gradient [9];	• Diffusion gradient of nutrients, waste, oxygen and drugs [9,16];
• Co-culture cannot create a microenvironment [17];	• Co-culture can mimic microenvironment [18];
• No resistance for anticancer drug [19];	• Resistant to anticancer drugs (mimic tumor morphology) [20].

**Table 2:** Comparison of drug testing results on 2D and 3D cultures.

Cell Cultures	Drugs	2D	3D
HCT-116 wt	5-FU, oxaliplatin, irinotecan, melphalan	equally and highly sensitive to 5-FU, oxaliplatin, irinotecan and melphalan	resistant or almost totally resistant to 4 standard drugs
HCT-116 wt/GFP			
NHEK	gefitinib	antiviral activity in concentrations too high for in vivo applications	gefitinib at concentration 0.5 µM was sufficient to induce meaningful reduction of replication and spreading of virus
SW1353	DXR, CIS, CQ	cell viability in 2D cultures were lower than in 3D cell cultures	cell viability in spheroid cultures were higher than in 2D cell cultures
	SAL	similar results for monolayers and 3D cell cultures	

## Abbreviations

2D	two-dimensional
3D	three-dimensional
AD	Alzheimer’s disease
APP	amyloid precursor protein
Aß	amyloid beta
Au	gold
CdTe	cadmium telluride
CGF	cowpox growth factor
CIS	cisplatin
CQ	chloroquine
ECM	extracellular matrix
EGFR	epidermal growth factor receptor
FAK	focal adhesion kinase
HD	Huntington’s disease
HTS	high-throughput screening
iPSCs	induced-pluripotent stem cells
NA	noradrenaline
NGF	nerve growth factor
NPs	nanoparticles
OPV	Orthopoxviruses
PAMAM	polyamidoamine
PCL	polycaprolactone
PD	Parkinson’s disease
PDMS	polydimethylsiloxane
PEG	polyethylene glycol
PLGA	poly(lactic-co-glycolic acid)
polyHEMA	poly(hydroxyethyl methacrylate)
PVA	polyvinyl alkohol
RGD	the tripeptide Arg-Gly-Asp consists of Arginine, Glycine, and Aspartate
SAL	salinomycin
WT	wild type
